# An Economic Analysis of Tourism Contribution for Urban Poverty Reduction in Major Tourist Towns of North Shewa Zone, Amhara Region, Ethiopia

**DOI:** 10.1155/2022/1116011

**Published:** 2022-09-30

**Authors:** Abebaw Hailu Fikire, Zemenu Bires, Girma Mulugeta Emeru

**Affiliations:** ^1^Department of Economics, Debre Berhan University, P.O. Box 445, Debre Berhan, Ethiopia; ^2^Department of Tourism and Hotel Management, Debre Berhan University, P.O. Box 445, Debre Berhan, Ethiopia

## Abstract

Tourism has a significant contribution to the livelihood diversification of the communities and supporting economic development efforts. This study aimed at an economic analysis of tourism's contribution to urban poverty reduction in Major Tourist Towns of North Shewa Zone, Amhara Region, Ethiopia. A multistage simple random sampling and purpose sampling were used to select sample respondents from the population in the study area. Hence, a total of 286 valid observations from 385 sample respondents were used for the analysis of the collected data. Both descriptive and inferential statistics were used to analyze the data. The finding of this study revealed that tourism has a significant and positive contribution to poverty reduction in urban areas, but the level of contribution is minimal. Besides, it exhibited that funding aids and government concern for tourism-targeted programs and access to credit infrastructure, tourism education, and policy were the significant factors that affect tourism's role in urban poverty reduction. Thus, to enhance its contributions and overcome industry bottlenecks, the government shall put tourism as a valuable development pillar, develop pro-poor strategies linking tourism with the urban poor, develop urban tourism development programs, and assign tourism-trained professional leaders to the sector.

## 1. Introduction

Tourism is among the fastest growing industries in terms of both revenue generation and employment creation [1]. Tourism is an increasingly important part of the global economy that is dependent on the annual movement of billions of travelers [2]. In 2019, the tourism sector experienced 3.5% growth outpacing the global economic growth of 2.5% for the 9^th^ consecutive year contributing 10.3% to the global GDP (USD 8.9 trillion) and creating around 330 million jobs, 1 in every 10 jobs all over the globe [3]. Similarly, the World Travel and Tourism Council 2019 report on travel and tourism impact exhibited that the share of tourism is about 10.4% of global GDP and 319 million jobs (i.e., 10% of the total employment) globally [4]. The city became the place of origin and at the same time a destination for an increasing number of tourists [5]. Economic growth is pro-poor and unbiased with equity but to be achievable requires the careful implementation of targeted macroeconomic policies on education and health, nutrition, and infrastructure [6]. Cities have a higher fitting territory, diversity, and quality of tourism products coming to fill a reach touristic ground, especially anthropogenic [5]. These all can be evidence for tourism that has valuable contributions to poverty reduction.

Tourism activities in urban areas such as the Olympic Games provide opportunities to host cities for speedy urban regeneration, stimulus growth, perk up transport and cultural facilities, and enhanced global recognition and prestige [7]. Besides, Olympic Games have been increasingly used as a trigger for a wide range of urban improvements, although there have been considerable variations in the scale of infrastructural investment and the public-private sector mix [8]. Tourism has also been found a significant contributor to livelihood diversification contributing more to urban households than rural communities [1].

Ethiopia possesses varied natural and cultural tourism resources where the majority of which are still underutilized [9, 10]. The causal relationship between international tourism and economic growth will have important implications for the development of different tourism marketing and policy decisions [11]. Thus, studying the role of tourism, particularly for urban economic change and poverty reduction, will be vital. The co-integration of tourism development and economic growth hypothesis of whether tourism growth to urban economic development or economic development leads to tourism growth needs a test in varied changes in time and place in the study area. This is because it has been revealed that economic development leads to international travel and an increase in tourism growth, but the reverse was found true in the previous study [11]. Tourism is a major group of international trade in services that boasted virtually uninterrupted growth demonstrating the strength and resilience of the sector (World Tourism Organization (UNWTO), 2017) [26]. Given that, various studies have been conducted at the country and regional levels, which have confirmed tourism's contribution to urban poverty reduction in Ethiopia. For instance, Dagnachew [13] conducted a study on community participation in tourism development in the Amhara Region evidence from Lalibela Town. Sebsibe [14] conducted a study on the role of tourism in economic growth, in the case of Ethiopia. Aynalem et al [15] conducted a study on employment opportunities and challenges in the tourism and hospitality sectors. Teshome et al. [[16]] conducted a study on the contribution of tourism to the Ethiopian economy and its impact on the GDP. Robinson and Jonker [17] conducted a study on tourism in Ethiopia: an urgent opportunity for economic diversification. Thus, this study is inspired to gain more insight into how tourism can reduce urban poverty in the major tourist area of North Shewa Zone, Amhara Region, Ethiopia. The contribution of the study to the existing literature has four points. First, some studies focused on its role in urban poverty giving focus on the general economic contribution of tourism [26] and economic impacts of tourism on small-scale enterprises [27], potentials and challenges of community-based tourism/ecotourism [9, 28] and policy planning tourism for food security and sustainable development in national parks [10], and tourism as a livelihood strategy for rural and urban livelihoods [1]. On the other hand, the contribution of tourism to reducing poverty in urban areas in Ethiopia and North Shewa remains untouched. Second, the economic contributions of tourism to urban poverty reduction remain uncovered. The tourism sector over the years has become an integral part of economic growth strategies and determinants for many developing and developed countries [21, 22], but this meaningful impact of tourism on the built environment is not yet uncovered. Third, the value and expected contributions of tourism to poverty reduction are not yet studied and analyzed other than the government reports. Finally, there is not enough study conducted on the economic analysis of tourism contribution to urban poverty reduction in selected urban tourism destinations of North Shewa Zone, Amhara Region, Ethiopia. Therefore, the objective of this study was to examine an economic analysis of tourism's contribution to urban poverty reduction in selected urban tourism destinations in the North Shewa Zone.

## 2. Literature Review

Tourism is the activity of persons traveling to and staying in places outside their usual environment for not more than one consecutive year for leisure, business, or any other purpose [23]. Tourism globally is the fastest growing industry in the world, bringing enhancement of job creation and bringing social and economic benefits to the individual, communities, and the government and private organizations that outweigh the general economic development and other sectors [24]. It plays a valuable role in the alleviation of poverty in the destination and has attracted many of its stakeholders to engage in the industry and support economic growth [25].

Poverty alleviation has been a focus of the sustainable development agenda since the 1990s [26] where the significance of tourism in poverty alleviation has also been assessed in Yuhu Village, China, where tourism cooperatives employed tourism as a livelihood strategy of the village. The study proved that the resources and power changes based on individual and collective levels are important providers of substantial improvements for the poor. The empowerment of locals in their status, legitimacy, and knowledge facilitated villagers to obtain favorable policy arenas and proved tourism cooperatives as niche tourism practical areas substantially improve the poverty level of the poor [27].

In slum tourism, areas of urban poverty become touristic attractions [28], where the urban poor experience miserable economic and social conditions and cope with adverse urban situations through strategies adopted mainly in their households [29]. Besides, slum tourism refers to guided tours of relatively poor neighborhoods that last up to four hours; they are often offered alongside other guided city tours by specialist guides and guiding firms [28, 30]. The extent to which tourism development has affected seven signifiers of poverty alleviation is examined based on accessibility improvement (transport and communication), prices of goods and services, entrepreneurial training, income-generating projects, employment opportunities, the general quality of life, and household income, which sound better to measure tourism effect for poverty alleviation [31]. Some developing countries such as Vietnam and Tanzania have thus embraced tourism as a tool of poverty alleviation [31, 32]. Apart from the positive contribution of tourism to poverty alleviation, it is criticized for its high-level leakage, low payment, and low participation of locals [27]. On the other hand, most of the local community felt that tourism development is having a positive impact and contributing to poverty alleviation, especially in terms of improving local facilities [31]. The positive effects of tourism on poverty alleviation are supported by statistics [33, 34]. Different from other economic activities that developed areas in competitiveness, several less developed regions also share advantageous positions in the tourism arena as worldwide popular destinations [27].

The impact of tourism on socioeconomic development and poverty alleviation can be felt in three key areas. First, since it can serve as a substantial source of foreign exchange earnings and public revenues, tourism contributes to economic development [35, 36]. Second, tourism activities are generally labor-intensive, so the expansion of these activities creates more employment opportunities for people of varying skills, including women [36, 37]. Third, tourism development generates better opportunities for residents to gain larger and more balanced benefits, when they participate fully in decision-making and ownership of tourism activities [38, 39]. In these ways, tourism can play an important role in the economic and sociocultural developments that are critical for poverty alleviation too [31].

Tourism can play in helping to address key development issues such as poverty, gender, trafficking of women, infrastructure, and the provision of health services [40]. Bayih and Singh [41] point out that in many instances the poor lack access to credit, which is essential in helping them to participate in the tourism economy. The poor very often had limited access to tourism infrastructure and assets. The governments in particular regions and communities lack essential market knowledge to allow them to develop pro-tourism strategies and products based on sound market information [42]. Generally, the empirical works that have been conducted show that tourism's contribution to urban poverty reduction is an important strategy to succeed in food security and improve the livelihood of society.

## 3. Materials and Methods

### 3.1. Description of the Study Area

North Shewa, one of the zonal administrations in the Amhara Region, takes its name from the kingdom or former province of Shewa. The administrative structure is divided into 22 woredas and 5 city administrations and has a population of 2, 226, 685. A total of 429,423 households were counted in this zone, which results in an average of 4.28 persons to a household and 413,235 housing units [54]. The three largest ethnic groups reported in North Shewa were the Amhara (90.73%), the Oromo (7.14%), and the Argobba (1.71%); all other ethnic groups made up 0.42% of the population (CSA (Central Statistical Agency), 2013). Amharic is spoken as a first language by 92.97%, and 6.32% spoke Oromiffa; the remaining 0.71% spoke all other primary languages reported. 94.71% of the population said they practiced Ethiopian Orthodox Christianity, and 4.91% were Muslim [55]. The study will be limited to Debre Berhan, Ankober, and Debre Sina, geographically selected based on pieces of information obtained from the North Shewa Zone Culture and Tourism Department as better tourist destinations.

### 3.2. Research Design and Approach

In this research, both descriptive research and explanatory research designs were employed based on a mainly cross-sectional design. The choice of the design is because descriptive design helps to expose and uncover the existing conditions based on the research formulated research hypothesis. Explanatory research design, on the other hand, helps to determine the impact relationship of various independent variables with the dependent variables and allows the manipulation of one or more independent variables [56]. These two designs are conclusive and help to make a conclusion and inferences about the general population based on the sample drawn. This study has used quantitative research approaches whereby the quantitative will help to quantify the data from the respondents about the issue under study. Besides, the qualitative research reports and concepts were used to triangulate the information obtained through the quantitative approach.

### 3.3. Source of Data and Method of Data Collection

The study employed both primary and secondary data sources. This primary data source comprises both the quantitative and qualitative tools to obtain from the primary data sources. The quantitative primary data source was a questionnaire. The data collection instruments were designed and developed customized from standardized questionnaires obtained from the literature. The questionnaires were designed as a closed-ended type. Hence, the structured questionnaires were developed based on a 5-point Likert scale and secondary sources such as reports and previous research articles were the research instruments. The questionnaires were pretested to check their clarity and understandability by local people (respondents) and to get quality data. The questionnaire was prepared in the English language and then translated into the local language (Amharic) to make it easy for communication. The questionnaires were distributed in a self-administered to each of the respondents, and respondents were informed to fill the questionnaire independently. Besides, the qualitative tools of data collection were used to get in-depth information through document analysis such as previous empirical findings and research reports about the issue under study. Thus, a structured questionnaire was the primary data source to collect data from households, whereas the secondary data sources were the previous research done, journals, articles, reports, and books, which were referred to make the findings of this study valid and reliable.

### 3.4. Sampling Design and Technique

The nature of this study requires the use of both purposive and random sampling techniques to make the data reliable and valid. Purposive sampling was employed for the selection of sample study sites. The random sampling technique was used to collect data from the household heads using a questionnaire survey. The selection of the sample urban households and respondents from relevant sectors of this research was based on a multistage random sampling technique. The subject of this study was the urban households at the destination who has an attachment to the tourist sites and tourism activities in the study area including community association members, micro, and small-scale business enterprises, city tour guides, and government culture and tourism offices at the destination in the study area. The members represented in the sample from each stratum were proportionate to the total number of elements in the respective strata [57].

### 3.5. Sample Size Determination

To determine the sample size of this study, the sample size determination formula for an unknown population, i.e., Cochran's correction formula of proportion, was used because the Cochran formula allows you to calculate an ideal sample size given a desired level of precision (± 5), desired confidence level (95%), and the estimated proportion of the attribute present in the population [58].

(1) $$\begin{array}{c} n_{0}=\frac{Z^{2}pq}{e^{2}}. \end{array}$$

Here, *n*_0_ is the sample size, *Z*^2^ is the normal curve that cuts off an area *α* at the tails 1−*α* equals the desired confidence level (95% in this study), *e* = (0.05) is the desired level of precision, *p* is the estimated proportion of an attribute that is present in the population, and *q* is 1−*p*. Thus, the total estimated sample size was calculated where the value of *Z* at a 95% confidence level is 1.96.

(2) $$\begin{array}{c} n_{0}=\frac{{\left(1.96\right)}^{2}\left(0.5\right)\left(0.5\right)}{{\left(0.05\right)}^{2}}=385. \end{array}$$

### 3.6. Method of Data Analysis

This study employed the Statistical Package for Social Sciences (SPSS version 23) software to analyze the data. In this study, descriptive and inferential statistics were used. Descriptive statistics were used to analyze the sociodemographic and economic characteristics of the respondents. In this study, inferential statistics such as correlations matrix, multiple linear regression, and ANOVAs were used for the quantitative data to examine the role of tourism in urban poverty reduction in the study area.

### 3.7. Reliability and Validity Analysis

The research has conducted the reliability and validity test to assure the appropriateness of the instrument and the consistency of the results using the pilot study. The validity of the research explains how well the collected data cover the actual area of investigation [59]. Hence, to confirm the validity of the instruments the research used standardized questionnaires and the items were reviewed by subject area experts. Sharma [60] classified the reliability statistics depending on the Cronbach alpha value: *α* ≥ 0.90 = excellent, 0.90 >*α* ≥ 0.80 = good, 0.80 > *α* ≥ .70 = acceptable, .70 > *α* ≥ 0.60 = questionable, 0.60 > *α* ≥ .50 = poor, and *α* < 0.50 = unacceptable. In this study, the reliability analysis was made by employing 58 observations, which are nearly 15% of the total sample population (i.e., 15%^*∗*^385 = 57.7) for a pilot survey conducted in Debre Berhan City. The reliability of the survey instruments was estimated based on the Cronbach alpha measure of internal consistency as indicated in the following table. The items from each of the constructs having very low inter-item correlation below.30 were removed. The reliability analysis revealed the Cronbach alpha coefficient that exhibited the consistency of the results ranging from .707 to .869, making the result acceptable based on Tavakol and Dennick [50] [42].  Table 1 presents the reliability analysis.

## 4. Results and Discussion

### 4.1. Descriptive Statistics

#### 4.1.1. Percentage of Administered Questionnaire

This study administered a total of 385 survey questionnaires where equal numbers of questionnaires were distributed to each of the three study sites in which 128 were distributed and an additional one was added to the survey questionnaire distributed to Debre Berhan City. From the total of 385 questionnaires distributed, 330 (85.71%) were returned. Though 85.71% of the total observations supposed to be distributed were collected, the final number of observations found filled, valid, and used for analysis accounted for 286 (86.67%) of 330 returned questionnaires and this number is 74.28% of the total sample observations.

#### 4.1.2. Respondent Characteristics

Table 2 shows that 160 (55.9%) of the respondents were males, whereas 126 (44.1%) of them were female respondents. As far as this study was concerned, of the respondents, the majority were youngsters under 18–35 years of age that accounting for 170 (59.4%) followed by ages between 36 and 45, which was about 80 (28.0%), and above 46 years that account for 36 (12.6%), respectively. The majority of the respondents were having a secondary school education level, which accounts for 89 (31.1%), followed by a college diploma (71; 24.8%) and a university degree and above, which accounts for 66 (23.1%), respectively. The remaining portion of sample respondents was found with an educational level of elementary school with the metric value of 16 (8.1%). The survey indicates that the majority of subjects of this study were found employed that accounting for 189 (66.1%) followed by unemployed residents with a total of 79 (27.6%) and pensioners that account for 18 (6.3%), respectively. Regarding household size, 149 (52.1%) of the respondents replied that their household numbers are between 4 and 6 members and 95 (33.2%) of them had a household size of less than 3 household members, whereas 27 (9.4%) and 15 (5.2%) of the respondents have a household size between 7 and 10 and more than 10 household members, respectively.

#### 4.1.3. Livelihood Strategy, Household Income, and Saving Characteristics

Table 3 shows the livelihood strategy people employed and the majority of the respondent led their household through self-employment (75; 26.2%) and government employment (59; 20.6%) followed by working being employed in private organizations (47; 16.4%), tourism and hotel (45; 15.7%), and engaging in trade (43; 15.0%), respectively. The respondents accounted for 13 (4.5%) and 4 (1.4%) are leading their lives by earning income through wages and being employed in nongovernmental organizations, respectively, in the sample sites.

The majority of the respondent's household heads earn an income between 1000 and 3000 birr (100; 35.0%) per month and a total monthly income of 3000–6000 birr (106; 37.1%). This was followed by the household size head monthly income and other household members' monthly income ranges of 3000–5000 birr (87; 30.4%), <3000 birr (99; 34.6%), <1000 birr (65; 22.7%), and 6000–10,000 birr (62; 21.7%), respectively. On the other hand, only 28 (9.8%) and 19 (6.6%) of the respondents got their income ranging from 5000 to 10,000 and > 10000 from the household head and other members of the household, respectively. The rest earned an income of >10,000 birr, which accounts for only 6 (2.1%). The income earned from the household head and other members was able to cover the costs of their expenditure, which accounted for 169 (59.1%) responses “Yes” and 117 (40.9%) replied “No.”

Another assessment of the study was family savings per month (birr), and the result revealed that the majority of the respondents have no saving pattern other than covering the whole costs of their household (140; 49.0%). Among the respondents, 77 (26.9%) had a monthly saving amount of <1000 birr followed by 1000–2000 birr, 2000–3000, and greater than 5000 birr that accounting for 40 (14.0%), 23 (8.0%), and 6 (2.1%), respectively.

### 4.2. Inferential Statistics

#### 4.2.1. Diagnostic Test

In this study, before running the regression analysis, different tests were tested. For instance, multicollinearity test for both continuous and discrete variables (variance inflation factor and correlation matrix), normality test (skewness and kurtosis), heteroscedasticity goodness-of-fit (F-statistic) test, and linearity test were seriously conducted for the multiple regression model.

#### 4.2.2. Relationship between Economic Impact of Tourism and Urban Poverty Reduction

The bivariate correlations run to explore the relationship between the economic impacts of tourism variable with urban poverty reduction. The correlation result revealed that the independent variable (tourism economic impact) has a significant correlation above 0.30 with the two dependent variables whereby correlation is significant at a .01 significance level with *r* = .502 for urban poverty reduction. The bivariate correlation analysis is presented in Table 4.

#### 4.2.3. Multiple Linear Regression Model

*(1). Role of Tourism for Urban Poverty Reduction*. The economic dimension of tourism impact indicators was employed to regress its impacts on urban poverty reduction. The model summary table also exhibited that tourism plays a significant role in its economic contributions to urban poverty reduction in the study areas accounting for about 25.2% of the variance in the urban poverty reduction variable. All in all, the result of this study revealed that tourism plays a vital role in enhancing urban poverty reduction efforts though it was found low in this study. Besides, the ANOVA table also revealed that the model employed to analyze the effect of tourism on urban poverty reduction was found significant with F-statistics of 95.776 and a significant value of 0.000 is a best-fitted model as far as the survey is concerned.

The model summary table also exhibited that tourism plays a significant role in its economic contributions for urban poverty reduction in the study areas accounting for about 25.2% of the variance in the urban poverty reduction variable. All in all, the result of this study revealed that tourism plays a vital role in enhancing the urban poverty reduction efforts though it was found low in this study (see Table 5). Besides, the ANOVA table also revealed that the model employed to analyze the effect of tourism on urban poverty reduction was found significant with F-statistics of 95.776 and a significant value of 0.000 is a best-fitted model as far as the survey is concerned.

The model coefficients exhibit the amount of unit change in efforts of urban poverty reduction due to a one-unit change in tourism practices to play by its economic impacts. This unit change in the dependent variable is represented by the beta value of 0.491 (Sig. = 0.000) attributed to a factor, i.e., urban poverty reduction significant at 0.01 and 0.05 levels of significance.  Table 6 presents the coefficient of determination of urban poverty reduction.

### 4.3. Factors Affecting Urban Poverty Reduction Efforts

The same scenario has been followed to identify the hindering factors of urban poverty reduction through tourism. Hence, the hindering the urban poverty reduction was employed to assess whether the elements or items affect the selected urban centers (Ankober, Debre Berhan, and Debre Sina) taken as sample sites for this study. Thus, to identify these factors, factor analysis with the principal component analysis was employed to extract the list of factors and to group each of the linear components onto each factor if found significant.

A total of 9 (nine) items or linear component factors (variables) were employed after checking the reliability of items in the pilot survey. Those variables were coded as 01—a lack of government programs targeted to the tourism informal sector in urban areas**; 02**—very little recognition of the potential of tourism development by aid agencies**; 03**—few officials working on poverty reduction have education or training in using tourism as a poverty reduction tool**; 04**—the poor lack access to credit in helping them to participate in the tourism economy; **05**—governments and NGOs lack the organizational capacity to respond to the opportunities provided by tourism development**; 06**—the poor very often have limited access to tourism infrastructure and assets**; 07**—outdated regulations make it impossible to develop innovative products and services**; 08**—governments and communities lack essential market knowledge to develop pro-tourism strategies and products**; and 09**—lack of necessary transportation and communications infrastructure essential to meeting the needs of the tourism industry.

The assumptions of relationship, randomness, and sampling adequacy were checked in the analysis of exploratory factor analysis (EFA).

The descriptive statistics revealed that all 9 linear component factors or variables have a mean value greater than 2.50. The deviation between 1.294 and 1.377 for a total of 286 valid observations was made for analysis. There were no missing data in the analysis. The measure of sampling adequacy is presented in Table 7.

The Kaiser–Meyer–Olkin (KMO) measure of sampling adequacy and Bartlett's test of sphericity also indicated that the sample size employed in this study was adequate and the assumption is met with KMO and Bartlett's test of sphericity value of 0.721 and Sig. = 0.000. A value varies between 0 and 1, where the value close to 1 indicates that patterns of correlations are relatively compact and so factor analysis should yield distinct and reliable factors. Kaiser [52] recommends accepting values greater than 0.5 are acceptable. Hence, the current value of KMO Bartlett's test of sphericity meets the assumption.

#### 4.3.1. Factor Extraction and Variance Explained

The present finding revealed that the total variance explained was 57.091%, which is attributed to the 3 factors extracted out of 9 linear component variables included in the model with eigenvalues greater than 1. Hence, from the table, the rotation sums of squared loadings indicated that the first factor contributed about 22.768% and the 2^nd^ contributes 17.534%, whereas the 3^rd^ factor accounted for 16.789% of the variance explained in urban poverty reduction. The cumulative percentage variance explained by the 3 factors was found to be 57.091% after an orthogonal rotation.  Table 8 presents the factor extraction and total variance explained.

#### 4.3.2. Factor Rotation

The rotated factor matrix indicates the rotated component matrix (also called the rotated factor matrix in factor analysis), which is a matrix of the factor loadings for each variable onto each factor. The values below 0.40 were suppressed while extracting the factors and are not displayed in the rotated component matrix where the factor loadings were sorted by size. The orthogonal rotation was used with the assumption that the variables are independent of each other [53]. Before rotation, most variables loaded highly onto the first factor (22.768% variance explained) followed by 17.534% and 16.789%, respectively. However, the rotation of the factor structure has clarified things considerably with the equivalence of variance explained. The suppression of loadings less than 0.4 and ordering variables by loading size was also made to make interpretation considerably easier.

As can be depicted in the rotated matrixcredit, infrastructure, tourism education, and policy factors have been extracted as a factor hindering the efforts made to enhance urban poverty reduction in the study areas of North Shewa. Three factors were extracted after rotation, the first factor accounted for 20.757% of the variance, the second factor accounted for 18.594%, and the third factor accounted for **17.374%** of the variance explained in the dependent variable, i.e., urban poverty reduction.

As it is indicated in the following table, the first factor consisted of four items that are related to access to credit infrastructure, tourism education, and policy (*TourEducPolcredit*). The second factor, which is a cluster of two variables, was related to access to communication infrastructure and market knowledge (*Mktkdge* Communication). Moreover, the third factor extracted encompasses three items that are related to lack of funding aids and government concern for tourism-targeted programs in urban areas (*Targetedtourprogram*). The rotated component matrix is presented in Table 9.

After the exploratory factor analysis was conducted and the three-component factors were extracted for constructs, namely, urban poverty reduction, the multiple linear regression was applied to identify and confirm which factors affect the role of tourism in urban poverty reduction in the study areas. The multiple linear regressions to confirm the extracted three factors for each of the two issues (urban poverty reduction) of component factors were explained and analyzed below.

#### 4.3.3. Confirmatory Factor Analysis Using Ordered Multiple Regression for Poverty Reduction

*(1) Regression Results for Factors Affecting Urban Poverty Reduction*. As indicated in Table 10, the model summary on the factors affecting urban poverty reduction shows the predicted variable; i.e., urban poverty reduction is explained by the introduced independent variables, namely, access to credit infrastructure, tourism education and policy, lack of access to communication infrastructure and market knowledge, and lack of funding aids and government concern for tourism-targeted programs for about **2.6%** with an adjusted *R* square value of 0**.26**. The variance explained in the model summary table is also supported by the coefficient table that exhibited some of the extracted factors that were significantly contributed to the urban poverty reduction efforts (see Table 10).

The coefficient result shows that the largest *β* value is the greatest predictor of urban poverty reduction. Among the independent variables, lack of funding aids and government concern for tourism-targeted programs was found a significant strongest factor affecting urban poverty reduction (*β* = −0.134, *p* < 0.05) followed by access to credit infrastructure, tourism education, and policy (*β* = 0.124, *p* < 0.05). Furthermore, there was no significant effect relationship between lack of access to communication infrastructure and market knowledge (*β* = −0.064, *p* > 0.05, i.e., .215) with the predicted variable, i.e., urban poverty reduction. Hence, the coefficients of determination table of the regression model revealed that lack of funding aids and government concern for tourism-targeted programs was found to be significant strongest factor affecting urban poverty reduction, and access to credit infrastructure, tourism education, and policy were significant factors that affect the urban poverty reduction efforts in the study areas.

Table 11 shows that the findings of this study revealed that lack of funding aids and government concern for tourism-targeted programs was found a significant strongest factor affecting urban poverty reduction (*β* = −0.134, *p* < 0.05) followed by access to credit infrastructure, tourism education, and policy (*β* = 0.124, *p* < 0.05), which were the significant factors that affect tourism's role for urban poverty reduction. Unlike the two factors, lack of communication infrastructure and market knowledge of the communities and the government was found insignificant factor proposed to be a factor affecting the urban poverty reduction efforts in the study areas of this study. Although the lack of promotion as a marketing tool in most of the tourism kinds of literature focusing on the Ethiopian tourism industry [9, 28, 54] before this research exhibited the significant challenge of a tourism industry that contradicts the present finding, another study found that marketing and promotion efforts are playing significantly positive for promoting tourism in playing its valuable role [55]. The present finding was supported by the study of Beza [9], which stated a low level of government attention and stakeholder participation, lack of financial support to the sector, lack of skilled manpower, and poor infrastructure as the major challenges of tourism in alleviating community-oriented problems such as poverty. These previous findings revealed that the low level of performance of the aforementioned issues results in a diminished contribution of tourism to urban poverty reduction. This demanded the counterbalance efforts of stakeholders to work in response to triggering factors. This study was also supported by previous studies, which revealed similar results [28, 54]. The present finding which shown lack of government concern and financial aids as significant challenges for tourism to play its role for poverty reduction was supported by the previous finding that exhibited budget pressures, financialconstraint, as well as governance issues, were the emerging issues and challenges for tourism [56]. The lack of proper tourism urban enhancement policy as a finding of this study was supported by another empirical finding that revealed that the success of the tourism industry is dependent on the proper programs and policies that most of the developing nations lack in the Caribbean [57].

#### 4.3.4. Discussion on the Role of Tourism in Urban Poverty Reduction

Tourism is one of the fastest growing industries in terms of both income generation and job creation [1]. It is an increasingly important part of the global economy that depends on billions of travelers' annual movement, often over large distances [2]. The sustainable livelihood framework emphasizes the interests of the communities and identifies the intricacy of people's lives [58]. Tourism development influences those traditional livelihood strategies that possess a valuable share of the entire communities' well-being [1, 59]. Similar to those findings, the present finding revealed that tourism contributes to about 25.2% of variance explained in the urban poverty reduction with the beta value *β* = 0.491 significant at 0.01 levels of significance. In line with this study, the study of Croes [60] revealed that tourism is an imperative determinant of poverty reduction. The emerging stage of development of tourism in the study areas makes a valuable contribution to tourism for poverty reduction. Similar to the present finding, the previous revealed that an increase in tourism development in terms of tourist demand and income generated leads to an increase in household welfare, which in turn leads to a decrease in poverty level [61]. Parallelly, the World Travel and Tourism Council 2019 report on travel and tourism impact exhibited that the share of tourism is about 10.4% of global GDP and 319 million jobs (i.e., 10% of the total employment) globally [4], which could enhance the poverty reduction efforts. The contribution of tourism to livelihood diversification was better predicted in the tourism enterprises and related organizations (TEs) who are mainly based in urban areas than the rural communities, which in turn indicates that the locals have lower participation than households from enterprises in tourism [1, 62]. The present results show that tourism has a positive and significant role in poverty reduction, and another study in Costa Rica and Nicaragua revealed that tourism's rate of poverty reduction was statistically significantly greater than that of agriculture for both countries [63]. This revealed that tourism has a meaningful potential to reduce poverty in national various rural, urban, and macro-level economies; hence, employing tourism as a poverty reduction tool is instrumental, especially for developing countries such as Ethiopia. Thus, tourism is playing for poverty reduction [55] for both rural and urban areas through the diversification of livelihoods [1], but its role is at its minimal stage [64]. Other findings that strengthen this study revealed that areas of urban poverty become touristic attractions in the form of slum tourism [28, 30], where the urban poor experience miserable economic and social conditions and cope with the adverse urban situations through strategies adopted mainly in their households [29]. Thus, it has been found that tourism could play a valuable role in urban poverty reduction efforts.

## 5. Conclusion and Recommendation

### 5.1. Conclusion

Tourism, among the largest industry in the world in terms of the creation of millions of new jobs and income, plays a significant role in the development of the urban economy so that it can contribute to poverty reduction in urban areas. This study aimed to analyze the economic contribution of tourism to urban poverty reduction in three selected towns of the North Shewa Zone, which are believed to have better tourist flow be either leisure, religious, or business name; Debre Berhan, Debre Sina, and Gorebela (Ankober). The study used purposive and random sampling techniques was employed to select 286 sample household heads and used a structured questionnaire. Descriptive and inferential statistics were employed to analyze the data. The study revealed that tourism was found to significantly and positively affect urban poverty reduction efforts. It has been shown that employing tourism as a tool for reducing urban poverty and enhancement of urban economic growth may be altered by many factors, which can either positively or negatively affect tourism goodwill. As can be seen from the findings, targeted programs and policies for tourism, funding aids, tourism education, proper tourism policy, and access to credit facilities were found determinant factors to determine the role of tourism in urban poverty reduction. In conclusion, urban areas, either large cities or small and medium towns, can employ tourism as an invaluable tool to support their efforts in bringing economic development and alleviating urban poverty. The nature of the tourism industry would also bring direct, indirect, and induced effects, various individuals, groups or families, and communities through employment, stimulating enterprises, and generating income through its effect are also altered by many financial and nonfinancial and policy and program determinant factors, which demands a serious and significant efforts to enhance its vital impacts on the people and the country at large.

### 5.2. Recommendations

This study suggested that the federal and regional governments shall take measures and set tourism among the priority development pillars to revitalize economic development and alleviate the urban poor. Therefore, good governance through strong sustainability institutions strengthens regulative mechanisms, processes, and organizational culture, which empower local urban communities. Besides, appropriate strategic planning policies are supposed to provide initiatives to promote sustainable tourism development in a way that tourism can bring valuable contributions to efforts for poverty reduction in urban areas. This policy should promote stakeholders' willing participation and awareness of sustainability issues and enhance technological innovation and infrastructure development. The hindering factors that triggered contributions of tourism to urban poverty reduction can be overthrown by employing tourism as a development economic tool by developing well-planned urban development strategy, linking the tourism sector with other sectors, and involving the urban communities. Therefore, to enhance its contributions and overcome industry bottlenecks, the government mainly shall put tourism as a valuable development pillar, develop pro-poor strategies linking tourism with the urban poor, and assign tourism-trained professional leaders to the sector.

## Figures and Tables

**Table 1:** Reliability analysis.

Variable	Cronbach's alpha	No. of items deleted	Total number of tested items
Factors hindering urban poverty reduction	0.707	0	9
Economic dimensions of tourism	0.852	1	8
Urban poverty reduction indicators	0.815	1	10

Source: Field Survey, 2021.

**Table 2:** Gender, age, education, employment status, and household size of the respondents.

Variable	Group	Frequency	Percent
Gender	Female	126	44.1
	Male	160	55.96

Age	18–35	170	59.4
	36–45	80	28.0
	Above 46	36	12.6

Education level	Elementary school	60	21.0
	Secondary school	89	31.1
	College diploma	71	24.8
	University degree and above	66	23.1

Employment status	Employed	189	66.1
	Unemployed	79	27.6
	Pensioner	18	6.3

Household size	<3	95	33.2
	4–6	149	52.1
	7–10	27	9.4
	>10	15	5.2

Source: Field Survey, 2021.

**Table 3:** Livelihood strategy, household income, and savings of respondents.

Means of livelihood			Average income/month/HH head (birr)			Average income/month/other members (birr)			Family saving per month (birr)		
	Freq.	%	Income/HH head	Freq.	%	Income level	Freq.	%	Level	Freq.	%
Trade	43	15.0	<1,000	65	22.7	<3000	99	34.6	No saving	140	49.0
Tourism and hotel	45	15.7	1,000–3,000	100	35.0	3000–6000	106	37.1	<1000	77	26.9
Govt. employee	59	20.6	3,000–5,000	87	30.4	6000–10000	62	21.7	1000–2000	40	14.0
Private employee	47	16.4	5,000–10,000	28	9.8	>10000	19	6.6	2000–5000	23	8.0
Self-employed	75	26.2	>10,000	6	2.1				>5000	6	2.1
Wage	13	4.5		Does your household monthly income cover your expenditure?							
NGO employed	4	1.4				Yes	169	59.1			
						No	117	40.9			

Source: Field Survey, 2021.

**Table 4:** Bivariate correlation analysis.

Correlations		
		Economic impacts of tourism
	Urban poverty reduction	0.502^*∗∗*^ (0.000)
N	286	

^*∗∗*^Correlation is significant at the 0.01 level (2-tailed). Source: Field Survey, 2021.

**Table 5:** Model summary table: urban poverty reduction.

Model summary^b^						
Model	R	R square	Adjusted *R* square	Std. error of the estimate	Durbin–Watson	
1	0.502^a^	**0.252**	0.250	0.839		1.914
			ANOVA^a^			
Model		Sum of squares	Df	Mean square	F	Sig.
1	Regression	67.469	1	67.469	95.776	0.000^b^
	Residual	200.063	284	0.704		
	Total	267.532	285			

^a^Dependent variable: urban poverty reduction. ^b^Predictors: (constant), Economic Impacts of Tourism. Source: Field Survey, 2021.

**Table 6:** Coefficient of determination: urban poverty reduction.

		Coefficients			*t*	Sig.	Collinearity statistics	
Model		Unstandardized coefficients		Standardized coefficients				
		*B*	Std. error	Beta			Tolerance	VIF
1	Constant	1.366	0.130		10.494	0.000		
	Economic impacts of tourism	0.491	0.050	0.502	9.787	0.000	1.000	1.000

Source: Field Survey, 2021. ^*a*.^Dependent variable: urban poverty reduction.

**Table 7:** KMO and Bartlett's test measure of sampling adequacy.

KMO and Bartlett's test		
Kaiser–Meyer–Olkin measure of sampling adequacy		0.721

Bartlett's test of sphericity	Approx. chi-square	439.983
	Df	36
	Sig.	0.000

Source: Field Survey, 2021.

**Table 8:** Factor extraction and total variance explained.

Total variance explained									
Component	Initial eigenvalues			Extraction sums of squared loadings			Rotation sums of squared loadings		
	Total	% of variance	Cumulative %	Total	% of variance	Cumulative %	Total	% of variance	Cumulative %
1	2.846	31.617	31.617	2.846	31.617	31.617	2.049	22.768	22.768
2	1.216	13.507	45.124	1.216	13.507	45.124	1.578	17.534	40.302
3	1.077	11.967	57.091	1.077	11.967	57.091	1.511	16.789	57.091
4	0.839	9.319	66.410						
5	0.803	8.922	75.332						
6	0.768	8.533	83.865						
7	0.545	6.053	89.918						
8	0.535	5.945	95.863						
9	0.372	4.137	100.000						

Source: Field Survey, 2021. Extraction method: principal component analysis.

**Table 9:** Rotated component matrix.

Rotated component matrix	Component			Communalities
	1	2	3	
Few officials working on poverty reduction have education or training in using tourism as a poverty reduction tool	0.771			0.617
The poor lack access to credit in helping them to participate in the tourism economy	0.679			0.522
Outdated regulations make it impossible to develop innovative products and services	0.624	0.484		0.640
The poor very often have limited access to tourism infrastructure and assets	0.575	0.474		0.556
Governments and communities lack essential market knowledge to develop pro-tourism strategies and products		0.709		0.503
Lack of necessary transportation and communication infrastructure essential to meeting the needs of the tourism industry		0.643		447
A lack of government programs targeting the tourism informal sector in urban areas			0.815	0.683
Very little recognition of the potential of tourism development by aid agencies	0.461		0.702	0.708
Governments and NGOs lack the organizational capacity to respond to the opportunities provided by tourism development		0.424	0.474	0.462
*Eigenvalues*	2.846	1.216	1.077	
*% of variance*	22.768	17.534	16.789	

Source: Field Survey, 2021. Extraction method: principal component analysis. Rotation method: varimax with Kaiser normalization. ^A^Rotation converged in 8 iterations.

**Table 10:** Model summary table: urban poverty reduction.

Model summary^b^					
Model	R	R square	Adjusted *R* square	Std. error of the estimate	Durbin–Watson
1	0.190^a^	0.036	0.026	0.996	1.591

Source: Field Survey, 2021. ^a^Dependent variable: UrbanPoverty_1. ^b^Predictors: (constant), lack of funding aids, a government concern, credit infrastructure, market knowledge, and communication infrastructure.

**Table 11:** Coefficients of determination: urban poverty reduction.

Coefficients								
Model		Unstandardized coefficients		Standardized coefficients	t	Sig.	Collinearity statistics	
		B	Std. error	Beta			Tolerance	VIF
1	Constant	2.873	0.238		12.048	0.000		
	Credit infrastructure, tourism education, and policy	0.123	0.055	0.146	2.239	**0.026**	0.798	1.252
	Lack of market knowledge and communication infrastructure	−0.064	0.052	−0.077	−1.241	0.215	0.897	1.115
	Lack of funding aids and government concern	−0.134	0.051	−0.171	−2.635	**0.009**	0.814	1.229

Source: Field Survey, 2021. ^a^Dependent variable: urban poverty reduction.

## Data Availability

The data that assist the findings of this can be accessed upon affordable request from the corresponding author.
